# Epithelial Cell Shedding and Barrier Function: A Matter of Life and Death at the Small Intestinal Villus Tip

**DOI:** 10.1177/0300985814559404

**Published:** 2015-05

**Authors:** J. M. Williams, C. A. Duckworth, M. D. Burkitt, A. J. M. Watson, B. J. Campbell, D. M. Pritchard

**Affiliations:** 1 Department of Gastroenterology, Institute of Translational Medicine, University of Liverpool, Liverpool, United Kingdom; 2 Norwich Medical School, University of East Anglia, Norwich Research Park, Norwich, United Kingdom

**Keywords:** genetically engineered mice, digestive tract, bacterial, immunologic, inflammation, nutritional, immunohistochemistry, imaging

## Abstract

The intestinal epithelium is a critical component of the gut barrier. Composed of a single layer of intestinal epithelial cells (IECs) held together by tight junctions, this delicate structure prevents the transfer of harmful microorganisms, antigens, and toxins from the gut lumen into the circulation. The equilibrium between the rate of apoptosis and shedding of senescent epithelial cells at the villus tip, and the generation of new cells in the crypt, is key to maintaining tissue homeostasis. However, in both localized and systemic inflammation, this balance may be disturbed as a result of pathological IEC shedding. Shedding of IECs from the epithelial monolayer may cause transient gaps or microerosions in the epithelial barrier, resulting in increased intestinal permeability. Although pathological IEC shedding has been observed in mouse models of inflammation and human intestinal conditions such as inflammatory bowel disease, understanding of the underlying mechanisms remains limited. This process may also be an important contributor to systemic and intestinal inflammatory diseases and gut barrier dysfunction in domestic animal species. This review aims to summarize current knowledge about intestinal epithelial cell shedding, its significance in gut barrier dysfunction and host-microbial interactions, and where research in this field is directed.

## The Small Intestinal Epithelium

The small intestinal epithelium allows water, electrolytes, and nutrients to be absorbed from the digesta while also functioning as an essential component of the gut barrier. This single-cell-thick epithelium prevents the entry of harmful microbes, toxins, and antigens from the intestinal lumen into the subjacent tissue, lymphatics, and vasculature. To permit efficient absorption, the small intestine has a thin and freely moveable nonadherent unstirred mucus layer to inhibit bacterial-epithelial interactions and diffusion of large molecules.^35^ Unlike other parts of the gastrointestinal tract, which have the additional protection of an inner adherent mucus layer,^35^ the mucus layer of the small intestine can be easily and rapidly disrupted in disease processes and conditions such as shock.^67^ The loss of epithelial contiguity can consequently result in serious perturbation of the gut’s barrier function.

**Figure 1.**
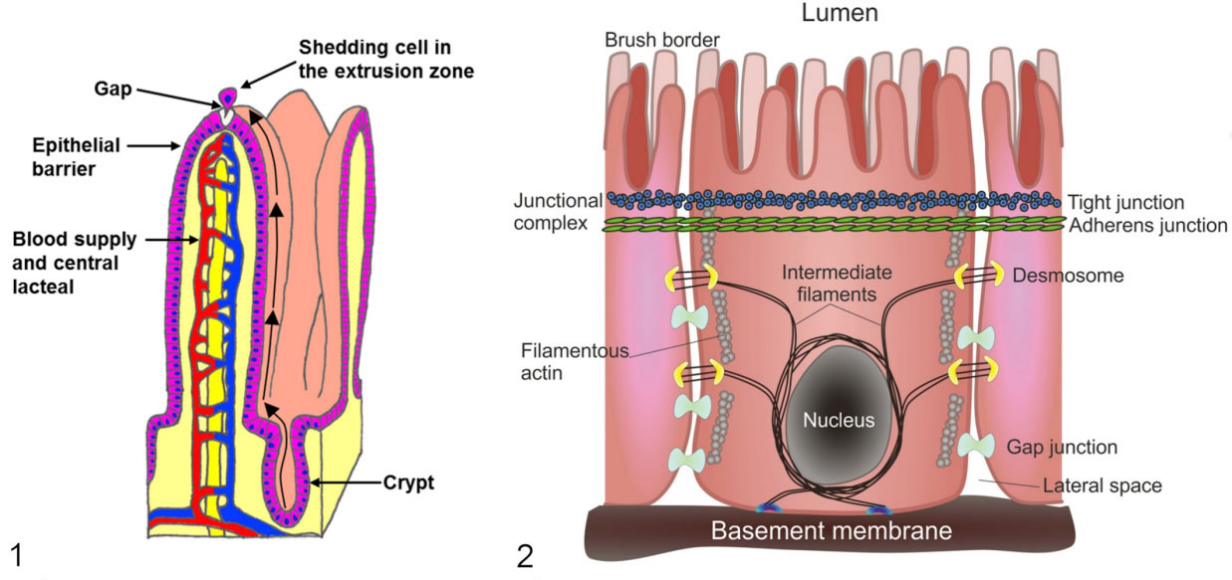
Illustration of small intestinal villi and epithelial cell turnover. New epithelial cells are generated in the crypt and migrate and differentiate during their journey along the villus until they are shed at the extrusion zone at the villus tip. **Figure 2.** Illustration of intestinal epithelial cell junctional complexes. Individual intestinal epithelial cells are joined to their neighbors by a continuous belt of tight junctions around the upper portion of the cell, which are responsible for gut/epithelial barrier function. The lateral spaces between cells allow paracellular transport.

A common feature conserved among mammals that possess small intestinal villi (the duck-billed platypus being an interesting exception that has intestinal folds)^40^ is that newly generated intestinal epithelial cells (IECs) within the crypt migrate toward the villus tip region, where loss of senescent epithelial cells occurs in the extrusion zone (Fig. 1). IECs have an extremely short lifetime. There is a rapid, almost complete renewal of the functional villus epithelium by the stem cells of the crypts of Lieberkϋhn every 2 to 6 days^50^ in most adult mammals. Enterocytes therefore have the highest turnover rate of any fixed-cell population in the body.^28^ Mathematical modeling suggests that in the mouse, an estimated 1400 mature enterocytes are shed from a single villus tip in each 24-hour period,^63^ equating to 2 × 10^8^ cells being shed from the small intestine per day. In humans, this daily loss has been estimated at 10^11^cells.^63^

Cell loss at the villus tip is, in normal circumstances, compensated for by stem cell mitosis within the crypts of Lieberkühn. The definitive identity of the intestinal stem cell, however, has been controversial for many years. Proposed stem cell models include the “+4 model” put forward by Marshman and colleagues^49^ and the “stem cell zone” model originally suggested by Cheng and Leblond.^13^ The “+4 model” hypothesizes that definitive stem cells occupy the fourth cell position when counting from the crypt base immediately above the Paneth cell zone. These putative stem cells positively label for B-cell lymphoma Mo-MLV insertion region 1 homolog (Bmi-1), homeodomain-only protein (Hopx), telomerase reverse transcriptase (Tert), and leucine-rich repeats and immunoglobulin-like domains protein 1 (Lrig1).^68^ In the “stem cell zone” model, it is postulated that the crypt base columnar (CBC) cells that are interspersed between Paneth cells are the true stem cells. In mice, in which intestinal cell dynamics have been best studied, 6 or more crypts^63^ provide each villus with new epithelial cells by asymmetric cell division of 14 to 16 CBCs at the crypt base.^65^ This CBC population, expressing leucine-rich repeat-containing G protein–coupled receptor 5 (Lgr5),^4^ produces transit-amplifying daughter cells that undergo migration along the crypt-villus axis and differentiate into absorptive enterocytes, goblet cells, or neuroendocrine cells*. In vivo* confocal imaging in mice has shown that central Lgr5-positive CBC cells maintain a position within the crypt base, while CBC cells in the upper part of the stem cell niche may be passively displaced into the transit-amplifying cell population.^65^ This conveyor belt from the crypt results in a continuous supply of epithelial cells, which on reaching the extrusion zone undergo apoptosis and are shed into the lumen. Paneth cells are an exception and either remain at the crypt base or migrate downward, depending on which hypothesis is true. Paneth cells possess prominent eosinophilic granules containing antimicrobial substances, including α-defensins and lysozyme,^8^ and have been hypothesized to protect and potentially regulate the stem cell niche. However, they are variably abundant among species, and while exceptionally abundant in some, such as the giant anteater,^79^ and prominent in primates, rodents, and the horse,^28^ they are absent in dogs, cats, and pigs,^8^ which raises questions about the role of these cells in this regard.

### The Epithelial Barrier

Intestinal epithelial cells are arranged as a single-cell-thick, tall columnar epithelium and possess a microvillus brush border at their apical plasma membrane. Microvilli are additionally covered by a matrix of glycoproteins constituting the glyocalyx and numerous digestive enzymes, including disaccharidases and aminopeptidases,^8^ which participate in the membranous phase of digestion. Individual epithelial cells are anchored at their basolateral pole to the basement membrane by hemidesmosomes and attached to their neighbors by a narrow continuous belt of tight junctions (Fig. 2). It is these tight junctions between epithelial cells that are responsible for maintaining epithelial barrier function (i.e. excluding intestinal luminal bacteria, noxious substances, and enzymes). However, the loss of whole cells from an epithelial monolayer presents a basic problem: how can cells detach without creating discontinuities and defects in the epithelial barrier?

The physiology of epithelial cell loss at the villus tip was historically viewed as a simple passive process of epithelial sloughing of individual cells or clusters of cells.^50^ In reality, intestinal epithelial cell shedding is a highly complex process of orchestrated events that maintains contiguity of the epithelium and gut barrier function. In species where it has been extensively studied, insights into the process of cell shedding have emerged. Understanding this process and its regulation, both in health and disease, is fundamental to understanding intestinal homeostasis.

Uncompensated enterocyte loss results in a decrease in the villus/crypt ratio. Major questions surround the control of migration and maturation of IECs and how and why apoptosis and shedding are restricted to the villus tip extrusion zone. Differences in the interaction between IECs and the basement membrane along the crypt-villus axis have been hypothesized, via gradients in expression of some basement membrane components^5^ or expression of some integrins by IECs.^5^ The balance of cell shedding at the villus tip matching renewal by the crypts is integral to maintaining small intestinal morphology and function. The major driving force of crypt proliferation is Wnt signaling.^78^ It has been known for many years that organs also secrete factors that inhibit proliferation, collectively termed *chalones*.^25^ Subsequent investigations have shown that in the small intestine, the most important of these factors that downregulate crypt epithelial production are probably bone morphogenic proteins (BMPs), which are generated within the intravillus mesenchyme.^78^ Many other factors also influence the rate of physiological cell loss at the villus tip and the rate of cell replacement from the crypts, including luminal nutrition,^10^ growth factors such as transforming growth factor β,^8^ hormones such as glucagon-like peptide 2,^8,10^ neural influences,^41^ circadian rhythms,^37^ and, importantly, the intestinal microbiota.^71^ In neonatal pigs, for example, prior to achieving climax flora, an IEC turnover of 7 to 10 days compares to a 2- to 3-day turnover when pigs are 3 weeks of age.^28^ This slower turnover rate occurs in germ-free animals, resulting in longer villi.^71^ Germ-free conditions also result in perturbation of nutrient absorption; mice reared in these conditions do not absorb monosaccharides or develop adipose reserves as efficiently as conventionally reared mice.^3^

IEC shedding as a process represents a very challenging phenomenon to study and understand. The small intestine undergoes extremely rapid postbiopsy and postmortem autolysis. Despite the high rate of physiological IEC loss from individual villi, the very short time it takes for individual cells to be extruded dictates that shedding events are observed relatively rarely in fixed specimens, even when fixation has been optimal. In fact, when hematoxylin and eosin (HE)–stained sections of the human small intestine were systematically examined, a shedding cell was observed in less than 6% of villus sections.^9^ Fixation by definition means that the dynamic process of extrusion cannot be fully appreciated; rather, various stages of the process may be observed without an obvious picture of the sequence of events.

Nonetheless, the morphological features associated with intestinal epithelial cell death, extrusion, shedding, and detachment have been studied in fixed tissues in several species and have revealed notable interspecies differences. In the guinea pig, apoptotic fragments are pinched off effete enterocytes, leaving junctional complexes intact between neighboring cells.^31^ In reindeer and seals, fragments of shedding enterocytes are lost either by extrusion or by phagocytosis by underlying macrophages.^55^ In cattle, γδ T cells are closely associated with apoptotic intestinal epithelial cells.^76^ Histologic studies in humans have shown shedding to be of the whole-cell extrusion type and that shed enterocytes are not associated with lymphocytes or macrophages.^9^ Therefore, the mechanism of epithelial shedding in humans appears morphologically similar to the process observed in mice, rats, and hamsters, suggesting that these species are appropriate models of human cell shedding and regulation.

### Whole-Cell Extrusion

**Figure 3.**
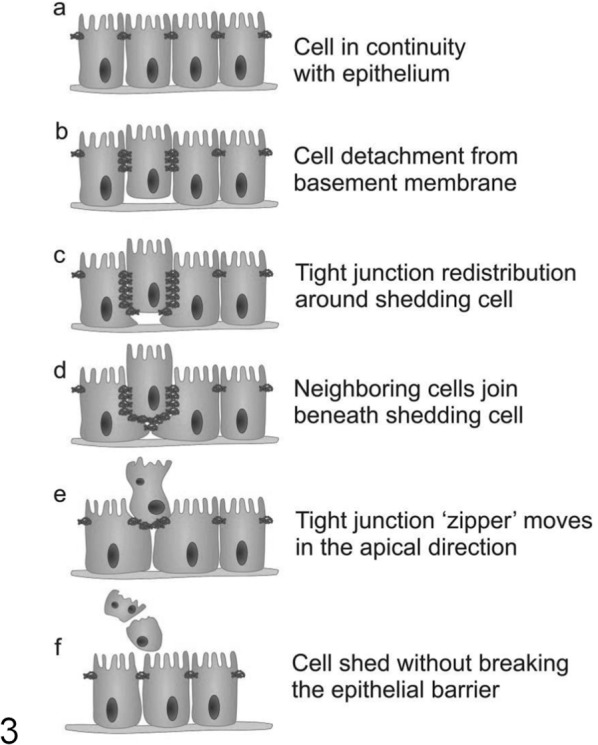
Illustration of the “zipper model” hypothesis of epithelial cell shedding. A complex sequence of orchestrated events (a–f) starts with detachment from the basement membrane and allows apical movement of an extruding epithelial cell. This is followed by rearrangement of tight junctions and advancement of lamellipodia underneath the extruding cell during and after the shedding process.

Several models have been proposed for the process of whole-cell extrusion of enterocytes from the villus. The “zipper model” is based on evidence from freeze-fracture transmission electron microscopy.^47^ Enterocytes undergoing extrusion exhibit basolateral movement of tight junctions down the plasma membrane shared with neighboring enterocytes (Fig. 3). Neighboring cells extend cytoplasmic processes underneath the shedding cell as it leaves the monolayer to reform tight junctions and maintain epithelial contiguity.^47^

**Figures 4–6.**
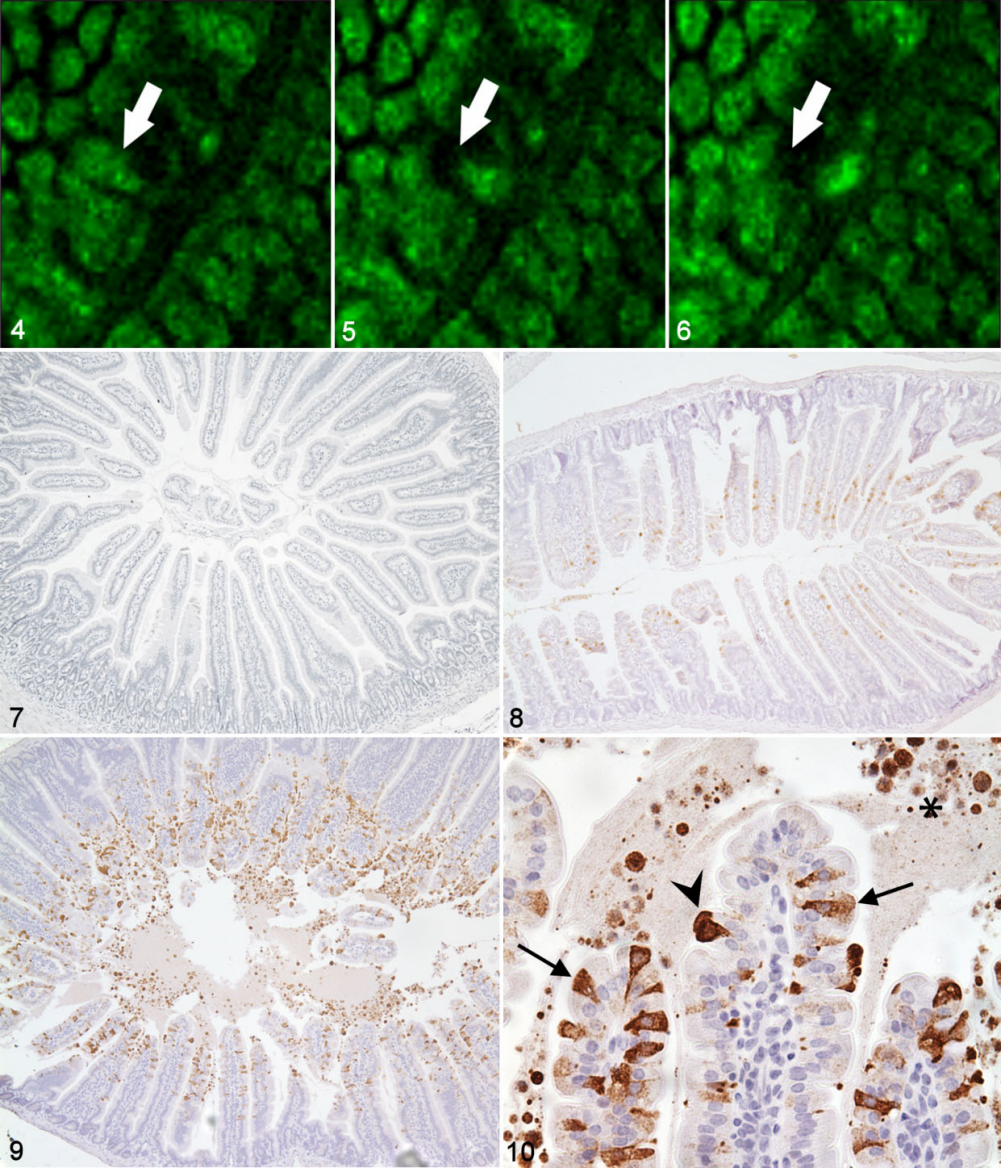
Small intestine; mouse. Lateral border of intestinal villus viewed en face by in vivo confocal imaging. Single epithelial cell nuclei undergoing physiological shedding (point of cell origin and resulting “gap” indicated by white arrows). Images taken at approximately 2.5-minute intervals in a terminally anaesthetized mouse. **Figures 7–10.** Small intestine; mouse. Pathological intestinal epithelial cell apoptosis and cell shedding induced by intraperitoneal injection of lipopolysaccharide (LPS). Active caspase 3 immunohistochemistry as a marker of apoptosis. **Figure 7**. Small intestine; mouse, untreated control. Negligible labeling of intestinal epithelial cells is observed. **Figure 8.** Small intestine; mouse. Increased intestinal epithelial cell labeling at 1 hour after LPS administration. **Figures 9–10.** Small intestine; mouse. A plateau of positive labeling of IECs is observed at 1.5 hours after LPS administration, with many individual positively labeled apoptotic cells in the epithelium (arrows) undergoing extrusion/cell shedding (arrowhead) into the intestinal lumen (asterisk). Acriflavine staining in Figs. 4 to 6. Envision^TM^-DAB with hematoxylin counterstain in Figs. 7 to 10.

In the context of physiological enterocyte shedding (Figs. 4–6), in vivo confocal microscopy studies of the small intestine in anaesthetized mice have shown that one of the first events in an enterocyte destined to undergo shedding is redistribution of tight junction protein zonula occludens 1 (ZO-1).^29^ This occurs approximately 15 minutes prior to cell shedding, first to the apical, then to the basolateral region of the shedding cell.^29^ Guan and colleagues^29^ observed that permeation of the membrane-impermeable marker Lucifer yellow only extends around the shedding cell as far as redistributed ZO-1 protein, supporting the critical nature of tight-junction proteins in maintaining the gut barrier. In 15% of these physiological shedding events, the neighboring cells were also shed within 5 to 10 minutes, suggesting that intercellular communication plays a role in coordinating group IEC shedding. IECs undergoing cell death and extrusion can be recognized at the villus tip by their expression of biochemical markers of apoptosis such as active caspase 3 and cleaved cytokeratin 18.^9^ It is not known whether cell death precedes the initiation of detachment or if it is detachment itself that causes cell death. In the latter scenario, the loss of anchorage-dependent survival signals from the underlying extracellular matrix and from neighboring cells results in a form of programmed cell death termed *anoikis*.

Fouquet and colleagues^23^ showed that in an ex vivo mouse small intestine model, early loss of E-cadherin, integral to adhering junctions in IECs, resulted in rapid execution of a Bcl-2 and caspase 9–dependent apoptotic pathway. Although a temporal relationship, rather than cause and effect, other groups have shown by active caspase 3 immunostaining that the apoptotic pathway appears to be activated prior to pathologic cell shedding and prior to IECs reaching the extrusion zone.^22,51,84^

## Pathological Epithelial Cell Shedding

In many intestinal diseases, cell loss from the villus exceeds the regenerative capacity of the crypts, due to epithelial injury to one or both components. Pathological epithelial cell shedding applies to an increased rate of villus epithelial cell apoptosis and extrusion in disease states. Acute loss of epithelial cells is followed by active villus core contraction,^54^ which effectively minimizes the area of denuded basement membrane to be reepithelialized.^54^ The remaining epithelial cells undergo flattening and attenuation to cover the exposed basement membrane, which otherwise may cause fusion of adjacent villi during restitution.^28^ Full restitution is dependent on a compensatory expansion of the crypts in the days following injury.^8^ Pathological epithelial cell shedding has been observed by in vivo confocal endomicroscopy in humans with inflammatory bowel disease (IBD),^38^ in mice following systemic tumor necrosis factor (TNF) administration,^39^ and during our own light microscopy studies of acute murine endotoxic shock (Figs. 7–10).^84^

A diverse range of stimuli and disease processes have been demonstrated to cause pathological epithelial cell shedding. These include TNF,^26,48,62^ bacterial lipopolysaccharide (LPS),^42,84^ indomethacin,^70^ the synthetic double-stranded viral RNA analogue (and Toll-like receptor 3 agonist) polyinosinic/polycytidylic acid,^51^ ischemia,^8^ ischemia-reperfusion injury,^33^ burn injury,^74^ trauma,^72^ increased lymphatic pressure,^44^ cocaine- or atropine-induced villus contraction,^44^ *Cryptosporidium parvum* infection,^22^ and toxins, such as that from *Bacteroides fragilis*.^85^

TNF, used extensively to induce pathological intestinal epithelial cell shedding, has provided valuable insights into the highly orchestrated events that lead to IEC extrusion and shedding in C57BL/6-Tg(Vil1-mRFP1/TJP1)#Tjr fluorescent-tagged ZO-1 transgenic mice.^29^ These studies have shown that one of the first events observed in cells destined to undergo shedding is the redistribution of ZO-1 to form a “funnel” around the apical cytoplasmic border of shedding cells. Along with other tight junction proteins, including claudins, the process extends to the basolateral border of the shedding cell completing extrusion.^29^

E-cadherin, F-actin, myosin II, Rho-associated kinase (ROCK), and myosin light chain kinase (MLCK) are redistributed during this highly dynamic process.^29^ Both ZO-1 redistribution and MLCK activation have been observed in neighboring enterocytes in a histologic study of cell shedding in humans.^9^ Marchiando et al^48^ showed that caspase activity, myosin motor activity (dynamin), and microtubule rearrangement are all required for shedding, thus refining the “zipper” model.^47^

It is unclear whether neighboring cells cooperate in aiding the shedding cell to exit the monolayer by forming an actin-myosin “purse string.” It has been suggested through in vitro observations in isolated villi that actin rearrangement takes place only in the enterocytes undergoing shedding and not in neighboring cells.^82^ This suggests that forces generated in the cell undergoing extrusion may be sufficient to complete the process and that the help of neighboring cells may not be necessary.

### The Role of TNF

TNF is probably a critical mediator for many stimuli of pathological epithelial cell shedding and acts via two known receptors: p55/TNFR1 or p75/TNFR2. TNF is synthesized as a pro-hormone; both secreted (17-kDa) and membrane-bound (18.5-kDa) forms exert biological effects.^69^ Membrane-bound pro-TNF is capable of juxtacrine signaling but can become solubilized by action of ADAM17,^6^ otherwise known as TACE (TNF-converting enzyme).

The cellular response to TNF is highly context dependent and varies by cell type, the relative expression of the 2 receptors, and downstream signaling responses. TNFR1 is expressed in a much wider variety of cell types than TNFR2; TNFR1 and TNFR2 are both present in the small intestinal epithelium.^43,73^

TNF is a potent stimulus of IEC shedding in mice.^48,62^ In histologic studies, intraperitoneal administration of TNF caused significant duodenal villus atrophy by 60 minutes postinjection (PI).^26,84^ Our studies of LPS-induced IEC shedding^84^ demonstrated that TNFR1 is a critical receptor for IEC apoptosis and cell shedding. Other studies have shown that TNFR1-deficient mice are resistant to the lethal effects of LPS and superantigen,^61^ potentially pointing to the importance of gut damage compared with other organ systems in the most acute phases of sepsis or LPS-induced mortality. The intestinal epithelium has been shown to be more sensitive to acute cytopathic changes and increased permeability than the pulmonary epithelium in an in vivo model of acute septic shock in cats and in human epithelia in response to inflammatory cytokines in vitro.^36^ These morphological alterations in intestinal epithelium may therefore reflect one of the earliest observable injuries in this type of inflammatory response, which may coincide with early gut barrier dysfunction.

TNF-induced IEC apoptosis and detachment in mice is mediated through TNFR1 and is independent of TNFR2 and p53.^62^ Following acute TNF administration, epithelial-specific expression of TNFR1 is necessary to induce apoptosis and shedding,^66^ rather than indirect vascular dysfunction causing ischemia/ischemia-reperfusion injury. This was shown by analyzing small intestinal IEC loss and apoptosis in a mouse model of acute TNF administration in which there is a conditional gain of function of the *Tnfr1* allele.^66^ These mice express TNFR1 within the intestinal epithelium rather than ubiquitously and have similar TNF-induced cell shedding as in wild-type mice. It is not clear if TNF delivered via the bloodstream or TNF generated by the epithelium is the most significant contributor to IEC apoptosis and shedding. It has however been shown that direct instillation of TNF into the duodenal lumen can cause intestinal damage in rats^34^ and that epithelial-specific dysregulated TNF production is highly important in driving epithelial damage.^30^

We reported that IEC apoptosis and cell shedding in response to LPS was lessened in TNFR2-deficient *Tnfrsf1b^tm1Imx^* homozygous mice,^84^ suggesting that this receptor plays a role in mediating apoptosis. Alternatively, the TNFR2 receptor may be responsible for suppressing TNF production, since markedly elevated amounts of TNF and increased pulmonary inflammation occur in these mice in response to LPS administration.^60^

### The Blood Supply of the Villus

In pathological epithelial cell apoptosis and cell shedding, the shedding process is not confined to the extrusion zone of the villus tip but extends further down the villus.^22,51,84^ However, the frequency of cell shedding still increases toward the villus tip. Hypoperfusion of the villus tip, particularly in pathological IEC shedding, has been hypothesized to be highly important in this effect.^7^ The vasculature of the villus is organized in such a way that venules run parallel and in very close proximity to the central arteriole, allowing countercurrent exchange of oxygen. This has important implications during hypoperfusion since when flow velocity decreases, more oxygen is transferred from the arteriole to the adjacent venules in the basal portion of the villus, potentially exacerbating the sensitivity of the villus tip to perfusion deficits.^81^ Interestingly, even in hypoxic conditions, there is increased production of TNF by intestinal epithelial cells,^77^ lending support to the notion that a diverse range of stimuli may converge on the TNF-TNFR1 pathway.

### LPS-Induced IEC Apoptosis and Shedding

Although the mechanisms of gut injury that occur in septic or endotoxic shock have not been fully established, it is thought to be initiated by the hypoxic and ischemic conditions brought about by blood maldistribution. In reality, it is likely to be a multifactorial process. Inducible nitric oxide synthase (iNOS), an important mediator of vasodilation and hemodynamics, has been strongly implicated in endotoxic shock-induced intestinal injury. It has been demonstrated that iNOS is responsible for rearrangement of tight junctions at later time points after LPS administration.^32^ Following LPS administration to cats, Crouser and coworkers^16^ reported intestinal epithelial necrosis at 2 hours PI; increased iNOS levels and apoptosis were observed only at 4 hours PI.^16^ During this study, ileal blood flow and oxygen saturation over the course of the experiment were relatively unchanged. The concept of gut mucosal damage being due to gut ischemia has also been challenged by comparing fluid resuscitation and superior mesenteric artery ligation in anaesthetized endotoxemic rabbits.^46^ Considered together with the resistance of TNFR1-deficient *Tnfrsf1a^tm1Imx^* homozygous mice to LPS-induced IEC apoptosis and shedding,^84^ and given that this process still occurs when functional TNFR1 is present only in intestinal epithelium,^66^ these findings suggest that gut injury in endotoxic shock is very dependent on epithelial responses rather than purely on blood flow alterations and blood oxygenation. Indeed, increased villus epithelial apoptosis occurs even when dysregulated TNF production is confined to the intestinal epithelium.^30^

### What Is the Significance of Pathological IEC Shedding?

The use of in vivo confocal microscopy has shown that after enterocytes are shed from the apex of the villus, epithelial discontinuities or “gaps” develop.^20,39,70,83^ These gaps contain residual ZO-1 left by the departing cell and are subsequently sealed by the process of neighboring epithelial cells re-forming tight junctions within 20 minutes.^29^ It has also been demonstrated that systemic TNF caused 20% of gaps to result in increased permeability and loss of barrier function.^39^

In the context of pathological epithelial cell shedding in the intestine of patients with IBD, it has been shown by confocal endomicroscopy that shedding events correlate with permeability defects and can aid in the prediction of disease relapse.^38^ It has also been shown that epithelial gap density correlates with disease severity in IBD.^45^ Additionally, there is a strong association of increased intestinal permeability in patients at high risk of developing intestinal disease.^52^ Although a cause-and-effect relationship is not established, this association suggests that increased gut permeability is important in the pathogenesis and progression of these conditions. Although increased intestinal permeability has been reported in intestinal diseases such as IBD,^12^ probably the best evidence that gut permeability can be directly implicated in the pathogenesis of intestinal disease has been described by Arrieta et al.^1^ They showed that interleukin 10 (IL-10)–deficient 129(B6)-Il10^tm1Cgn^ mice developed increased small intestinal permeability prior to developing spontaneous colitis. When these mice were administered AT-1001, a synthetic peptide that blocks the zonulin receptor leading to reduced small intestinal permeability, the mice had a significant reduction in the severity of colitis.

Our studies of LPS-induced apoptosis and cell shedding in mice suggest that increased gut permeability and clinical diarrhea temporally correlate with IEC shedding.^84^ Although these data imply fluid loss into the gut lumen, it is not until later time points, after the discontinuation of IEC shedding, that increased gut to circulation permeability occurs. The temporal change in fluid and large molecular movement may, however, reflect changing tonicity of the gut content as it has been demonstrated that hypotonicity of the luminal content favors the inward permeation of solutes.^38^

**Figure 11.**
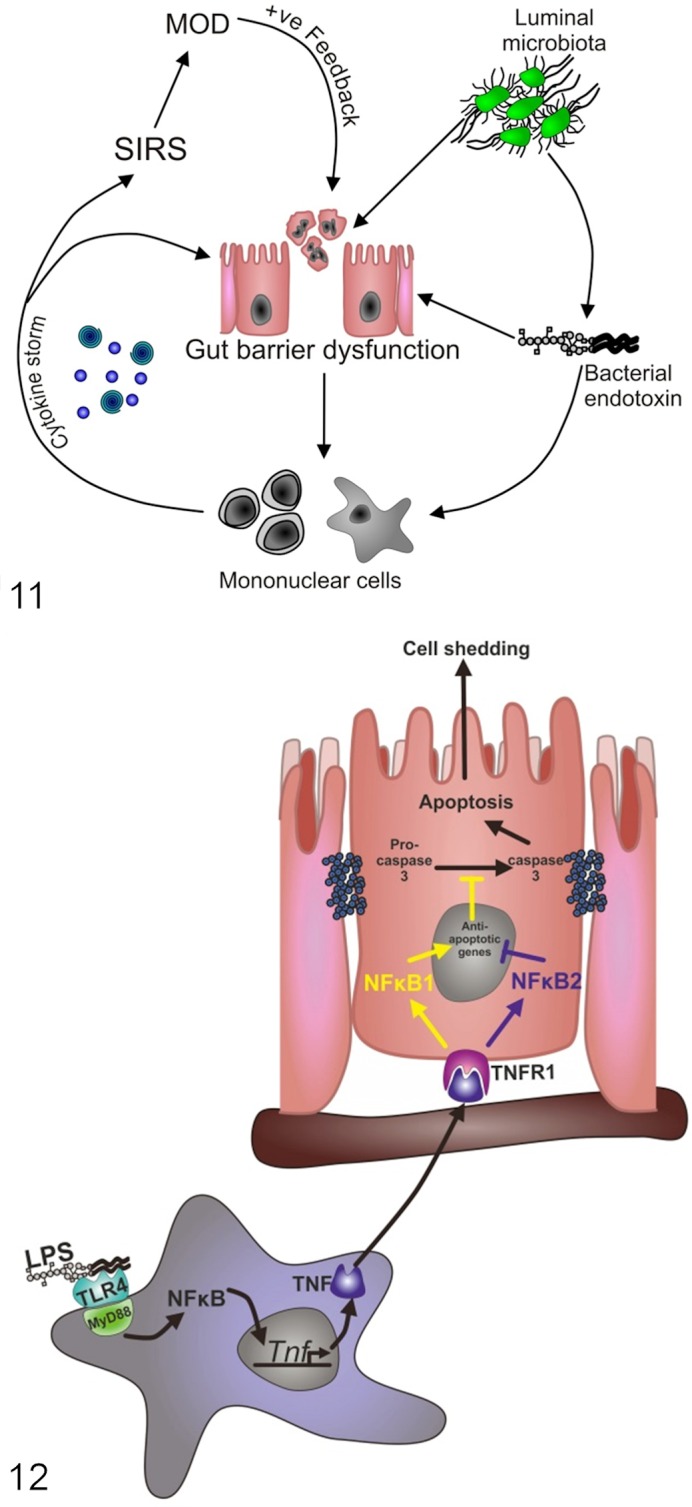
Diagram outlining the gut origin of sepsis hypothesis. Microbes or microbial products such as lipopolysaccharide/bacterial endotoxin translocate across the gut barrier and result in mononuclear cell activation and cytokine release, which worsens gut barrier function. This may result in a “cytokine storm,” systemic inflammatory response syndrome (SIRS), and multiple-organ dysfunction (MOD) in a positive feedback loop. **Figure 12.** Diagram summarizing a potential mechanism by which lipopolysaccharide (LPS) induces apoptosis in intestinal epithelial cells. TLR4-expressing mononuclear cells (monocytes/macrophages/dendritic cells) recognize systemic LPS and produce tumor necrosis factor (TNF). TNF is released into the systemic circulation and binds with TNFR1 on intestinal epithelial cells, triggering apoptosis and shedding if *Nfkb2* encoded protein (p100/p52) signaling dominates or cell survival if *Nfkb1* encoded protein (p105/p50) signaling dominates.

The gut is an extremely large reservoir of bacteria, including LPS-containing Gram-negatives. Gut barrier dysfunction induced by shock has therefore led to the development of the “gut origin of sepsis” hypothesis. This hypothesis proposes that shock initiates failure of the gut barrier, which in turn allows bacteria and/or endotoxin into the circulation in a positive feedback loop^27^ (Fig. 11). Previous studies have shown extensive gut injury with crypt apoptosis several hours after the induction of endotoxic or septic shock.^14,15,30^ Increased intestinal permeability has been shown in the ileum of rats as early as 2 hours after LPS administration, and in mice, bacterial translocation has been detected 24 hours post-LPS administration.^19^

Some pathogenic bacteria and viruses are known to undergo intestinal invasion via M cells.^28,53^ However, it also has been observed by electron microscopy that *Salmonella enterica* serovar Typhimurium DT104 invades the crevices created during early epithelial cell extrusion at the villus tip in experimental infection in pigs.^53^ Ex vivo studies have also demonstrated that the pathogenic bacterium *Listeria monocytogenes* invades sites of epithelial cell extrusion at the villus tip in experimental infection in rabbits.^59^ E-cadherin, which is usually inaccessible beneath tight junctions, has been shown to be exposed at extrusion sites and epithelial gaps.^48^ In the case of *L. monocytogenes*, invasion is mediated via bacterial internalin A and host E-cadherin interaction.^57,59^ It is therefore postulated that intestinal epithelial gaps and microerosions at the villus tip may also be exploited as portals of entry for commensal bacteria.

### The Influence of NF-κB Signaling

*Nuclear factor kappa-light-chain-enhancer of activated B cells* (NF-κB) is the umbrella term for a family of highly evolutionarily conserved cytosolic proteins that are important in preserving intestinal epithelial integrity in response to inflammatory stimuli.^24,30^ These proteins, when activated, are released from their specific inhibitor of κB (IκB) protein, dimerize in various combinations, and oscillate into and from the nucleus in an energy-dependent manner.^56^ In the nucleus, NF-κB proteins act as transient transcription factors controlling the expression of a large array of genes, including those involved in regulating inflammation, cell stress, cell adhesion, and crucially in the context of intestinal epithelial cells, cell death, or survival.

Mice lacking the p65/RelA NF-κB subunit in IECs exhibit profoundly increased IEC apoptosis, have dysregulated IEC proliferation, and suffer spontaneous intestinal failure with loss of the crypt-villus architecture.^75^ Nuclear translocation of p65/RelA occurs in villus IECs that resist shedding in a *Cryptosporidium parvum* model of IEC shedding in piglets.^22^

Our^84^ and other studies have shown that transgenic mice with specific NF-κB family member deletion^24^ or alterations in NF-κB activation pathways^30^ have dramatically altered susceptibility to acute stimuli of small intestinal injury. NF-κB appears to be important in dictating the fate of the epithelial cell in response to TNF or LPS, with p105/p50 signaling favoring cell survival and p100/p52 favoring apoptosis (Fig. 12).^84^ The mechanisms responsible for these interactions are however incompletely understood.

## Future Directions and Concluding Remarks

Many questions remain regarding how pathological IEC apoptosis and shedding affect gut permeability and what the immediate and longer term consequences of increased gut permeability may be. Acute epithelial defects appear to be associated with villus contraction and initial fluid exudation into the gut lumen, followed later by the movement of large molecules out of the gut lumen and into the bloodstream. The most obvious detrimental outcome in acute inflammatory responses appears to be the serious derangement of fluid and electrolyte balance, with loss of albumen into the gut lumen.^24^ This may be followed by bacterial translocation.^18^ These events constitute failure of the gut barrier, often concurrently with multiple-organ failure. Due to the complexity of the terminal sequence of events in shock that culminate in disseminated intravascular coagulation (DIC) and death, it is difficult to separate cause and effect. However, in less complex organisms, such as the fruit fly *Drosophila melanogaster*, with its simpler organ and circulatory systems, failure of the gut barrier is a predictor of imminent death of the organism.^64^

In more chronic situations, increased gut permeability and exposure of subepithelial tissues to gut luminal antigens may trigger inappropriate immune and inflammatory responses. Diet also seems to play an important role in regulating intestinal permeability. High-fat diets cause increased gut permeability and metabolic endotoxemia, as well as increased circulating LPS, originating from the gut,^58^ with low-grade inflammation.^11^ Chronic metabolic endotoxemia, when artificially induced in mice on a normal diet, has been shown to initiate obesity and insulin resistance,^11^ similar to if these animals are fed a high-fat diet.^11^ A high-fat diet in mice also increases the amount of Gram-negative bacteria within the gut and promotes LPS translocation across the intestinal epithelium.^11^ Mounting evidence suggests that epithelial responses and intestinal permeability may be highly modulated by the gut microbiota.^2,21^ Host-microbial interactions likely play a significant role in regulating intestinal epithelial apoptosis and cell shedding.

IEC apoptosis and cell shedding probably occur in a variety of clinical scenarios in which there are surges of TNF. Many patients with IBD are given anti-TNF antibody treatment, but this is extremely costly, and unfortunately, a proportion of patients either fail to respond or become refractory.^17^ Chronic progressive idiopathic intestinal diseases in animals may also share a TNF-based pathogenesis. TNF is integral to effective leukocyte-mediated immune responses, and preventing its signaling, although an improvement on the more nonselective immune suppression therapy with corticosteroids, results in defective immune function. A better understanding of how TNF production affects the epithelial as opposed to the immune cell compartments is required to allow more targeted treatment approaches. Targeting regulatory elements downstream of TNF receptors, such as NF-κB signaling, may also allow for more sophisticated therapeutic strategies to be developed that prevent pathological intestinal epithelial cell apoptosis and cell shedding, thereby enhancing barrier integrity.

Intriguingly, the epithelial cell compartment appears to be an important source of TNF production initiating enterocyte apoptosis and cell shedding. Transcriptional and posttranscriptional control is critical in determining the outcome of excessive TNF production.^30^ Modulating this proinflammatory cytokine via TACE, mitogen-activated protein kinase,^30^ or matrix metalloproteinase 13^80^ may therefore represent alternative strategies for preventing pathological intestinal epithelial cell shedding.

Investigating the process of intestinal epithelial cell death and the key initiating and regulatory factors may further our understanding of the pathogenesis of idiopathic inflammatory intestinal diseases and possibly intestinal cancers. It may also allow prevention of gut barrier dysfunction in intestinal and systemic disease states. Addressing and understanding the mechanisms of gut barrier dysfunction may allow future development of therapeutic and prophylactic strategies for the prevention of the serious sequelae of bacterial translocation, sepsis, and endotoxemia in human and animal medicine.

## References

[1] Arrieta MC Madsen K Doyle J Reducing small intestinal permeability attenuates colitis in the IL10 gene-deficient mouse. Gut. 2009;58 (1):41–48. 18829978 10.1136/gut.2008.150888 PMC2597688

[2] Aziz Q Doré J Emmanuel A Gut microbiota and gastrointestinal health: current concepts and future directions. Neurogastroenterol Motil. 2013;25 (1):4–15. 23279728 10.1111/nmo.12046

[3] Backhed F Ding H Wang T The gut microbiota as an environmental factor that regulates fat storage. Proc Natl Acad Sci USA. 2004;101 (44):15718–15723. 15505215 10.1073/pnas.0407076101 PMC524219

[4] Barker N van Es JH Kuipers J Identification of stem cells in small intestine and colon by marker gene Lgr5. Nature. 2007;449 (7165):1003–1007. 17934449 10.1038/nature06196

[5] Beaulieu JF Differential expression of the VLA family of integrins along the crypt-villus axis in the human small intestine. J Cell Sci. 1992;102 (3):427–436. 1506425 10.1242/jcs.102.3.427

[6] Black RA Rauch CT Kozlosky CJ A metalloproteinase disintegrin that releases tumour-necrosis factor-alpha from cells. Nature. 1997;385 (6618):729–733. 9034190 10.1038/385729a0

[7] Blikslager AT Life in the gut without oxygen: adaptive mechanisms and inflammatory bowel disease. Gastroenterology. 2008;134 (1):346–348. 18166362 10.1053/j.gastro.2007.11.049

[8] Brown CC Baker DC Barker IK Alimentary system. In: Maxie MG ed. Pathology of Domestic Animals. 5th ed Edinburgh, UK: Elsevier Saunders; 2007:69–78.

[9] Bullen TF Forrest S Campbell F Characterization of epithelial cell shedding from human small intestine. Lab Invest. 2006;86 (10):1052–1063. 16909128 10.1038/labinvest.3700464

[10] Burrin DG Stoll B Guan X Glucagon-like peptide 2 dose-dependently activates intestinal cell survival and proliferation in neonatal piglets. Endocrinology. 2005;146 (1):22–32. 15486229 10.1210/en.2004-1119

[11] Cani PD Amar J Iglesias MA Metabolic endotoxemia initiates obesity and insulin resistance. Diabetes. 2007;56 (7):1761–1772. 17456850 10.2337/db06-1491

[12] Casellas F Aguade S Soriano B Intestinal permeability to Tc-99m-diethylenetriaminopentaacetic acid in inflammatory bowel-disease. Am J Gastroenterol. 1986;81 (9):767–770. 3529937

[13] Cheng H Leblond CP Origin, differentiation and renewal of the four main epithelial cell types in the mouse small intestine: V. Unitarian theory of the origin of the four epithelial cell types. Am J Anat. 1974;141 (4):537–561. 4440635 10.1002/aja.1001410407

[14] Coopersmith CM Stromberg PE Davis CG Sepsis from *Pseudomonas aeruginosa* pneumonia decreases intestinal proliferation and induces gut epithelial cell cycle arrest. Crit Care Med. 2003;31 (6):1630–1637. 12794397 10.1097/01.CCM.0000055385.29232.11

[15] Coopersmith CM Stromberg PE Dunne WM Inhibition of intestinal epithelial apoptosis and survival in a murine model of pneumonia-induced sepsis. JAMA. 2002;287 (13):1716–1721. 11926897 10.1001/jama.287.13.1716

[16] Crouser ED Julian MW Weinstein DM Endotoxin-induced ileal mucosal injury and nitric oxide dysregulation are temporally dissociated. Am J Respir Crit Care Med. 2000;161 (5):1705–1712. 10806178 10.1164/ajrccm.161.5.9907043

[17] de Silva PSA Nguyen DD Sauk J Long-term outcome of a third anti-TNF monoclonal antibody after the failure of two prior anti-TNFs in inflammatory bowel disease. Aliment Pharmacol Ther. 2012;36 (5):459–466. 22784296 10.1111/j.1365-2036.2012.05214.x

[18] Deitch EA Berg R Specian R Endotoxin promotes the translocation of bacteria from the gut. Arch Surg. 1987;122 (2):185–190. 3545142 10.1001/archsurg.1987.01400140067008

[19] Deitch EA Specian RD Berg RD Endotoxin-induced bacterial translocation and mucosal permeability: role of xanthine oxidase, complement activation, and macrophage products. Crit Care Med. 1991;19 (6):785–791. 2055056 10.1097/00003246-199106000-00010

[20] Duckworth CA Watson AJ Analysis of epithelial cell shedding and gaps in the intestinal epithelium. Methods Mol Biol. 2011;763:105–114. 21874447 10.1007/978-1-61779-191-8_7

[21] Everard A Belzer C Geurts L Cross-talk between *Akkermansia muciniphila* and intestinal epithelium controls diet-induced obesity. Proc Natl Acad Sci USA. 201 3;110 (22):9066–9071. 10.1073/pnas.1219451110 PMC3670398 23671105

[22] Foster DM Stauffer SH Stone MR Proteasome inhibition of pathologic shedding of enterocytes to defend barrier function requires X-linked inhibitor of apoptosis protein and nuclear factor kappaB. Gastroenterology. 2012;143(1):133–144.e4. 22446197 10.1053/j.gastro.2012.03.030

[23] Fouquet S Lugo-Martinez VH Faussat AM Early loss of E-cadherin from cell-cell contacts is involved in the onset of Anoikis in enterocytes. J Biol Chem. 2004;279 (41):43061–43069. 15292248 10.1074/jbc.M405095200

[24] Gadjeva M Wang Y Horwitz BH NF-κB p50 and p65 subunits control intestinal homeostasis. Eur J Immunol. 2007;37 (9):2509–2517. 17705134 10.1002/eji.200737186

[25] Gamer LW Nove J Rosen V Return of the Chalones. Dev Cell. 2003;4 (2):143–144. 12586054 10.1016/s1534-5807(03)00027-3

[26] Garside P Bunce C Tomlinson RC Analysis of enteropathy induced by tumour necrosis factor alpha. Cytokine. 1993;5 (1):24–30. 8485304 10.1016/1043-4666(93)90020-6

[27] Gatt M Reddy BS Macfie J Review article: bacterial translocation in the critically ill—evidence and methods of prevention. Aliment Pharmacol Ther. 2007;25 (7):741–757. 17373913 10.1111/j.1365-2036.2006.03174.x

[28] Gelberg HB Alimentary system. In: McGavin MD Zachary JF eds. Pathologic Basis of Veterinary Disease. 4th ed St Louis, MO: Elsevier Mosby; 2007:342–360.

[29] Guan Y Watson AJ Marchiando AM Redistribution of the tight junction protein ZO-1 during physiologic shedding of mouse intestinal epithelial cells. Am J Physiol Cell Physiol. 201 1;300 (6):C1404–C1414. 21346149 10.1152/ajpcell.00270.2010 PMC3118625

[30] Guma M Stepniak D Shaked H Constitutive intestinal NF-kappaB does not trigger destructive inflammation unless accompanied by MAPK activation. J Exp Med. 2011;208 (9):1889–1900. 21825016 10.1084/jem.20110242 PMC3171091

[31] Han H Iwanaga T Fujita T Species-differences in the process of apoptosis in epithelial cells of the small intestine: an ultrastructural and cytochemical study of luminal cell elements. Arch Histol Cytol. 1993;56 (1):83–90. 8499128 10.1679/aohc.56.83

[32] Han X Fink MP Yang R Increased iNOS activity is essential for intestinal epithelial tight junction dysfunction in endotoxemic mice. Shock. 2004;21 (3):261–270. 14770040 10.1097/01.shk.0000112346.38599.10

[33] Ikeda H Suzuki Y Suzuki M Apoptosis is a major mode of cell death caused by ischaemia and ischaemia/reperfusion injury to the rat intestinal epithelium. Gut. 1998;42 (4):530–537. 9616316 10.1136/gut.42.4.530 PMC1727054

[34] Jackson GDF Dai W Sewell WA Bile mediates intestinal pathology in endotoxemia in rats. Infect Immun. 2000;68 (8):4714–4719. 10899877 10.1128/iai.68.8.4714-4719.2000 PMC98417

[35] Johansson ME Sjovall H Hansson GC The gastrointestinal mucus system in health and disease. Nat Rev Gastroenterol Hepatol. 2013;10 (6):352–361. 23478383 10.1038/nrgastro.2013.35 PMC3758667

[36] Julian MW Bao S Knoell DL Intestinal epithelium is more susceptible to cytopathic injury and altered permeability than the lung epithelium in the context of acute sepsis. Int J Exp Pathol. 2011;92 (5):366–376. 21838744 10.1111/j.1365-2613.2011.00783.x PMC3174335

[37] Kaur P Potten CS Circadian variation in migration velocity in small intestinal epithelium. Cell Tissue Kinet. 1986;19 (6):591–599. 3802183 10.1111/j.1365-2184.1986.tb00760.x

[38] Kiesslich R Duckworth CA Moussata D Local barrier dysfunction identified by confocal laser endomicroscopy predicts relapse in inflammatory bowel disease. Gut. 2012;61 (8):1146–1153. 22115910 10.1136/gutjnl-2011-300695 PMC3388727

[39] Kiesslich R Goetz M Angus EM Identification of epithelial gaps in human small and large intestine by confocal endomicroscopy. Gastroenterology. 2007;133 (6):1769–1778. 18054549 10.1053/j.gastro.2007.09.011

[40] Krause WJ Intestinal mucosa of the platypus, Ornithorhynchus anatinus. Anat Rec. 1975;181 (2):251–265. 10.1002/ar.1091810207 1115355

[41] Lachat J-J Gonçalves R Influence of autonomic denervation upon the kinetics of the ileal epithelium of the rat. Cell Tissue Res. 1978;192 (2):285–297. 699017 10.1007/BF00220746

[42] Lai CW Sun TL Lo W Shedding-induced gap formation contributes to gut barrier dysfunction in endotoxemia. J Trauma Acute Care Surg. 2013;74 (1):203–213. 23271096 10.1097/TA.0b013e3182788083

[43] Lau KS Juchheim AM Cavaliere KR In vivo systems analysis identifies spatial and temporal aspects of the modulation of TNF-alpha-induced apoptosis and proliferation by MAPKs. Sci Signal. 2011;4(165):ra16. 21427409 10.1126/scisignal.2001338 PMC3963028

[44] Lee JS Epithelial cell extrusion during fluid transport in canine small intestine. Am J Physiol. 1977;232 (4):E408–E414. 851184 10.1152/ajpendo.1977.232.4.E408

[45] Liu JJ Madsen KL Boulanger P Mind the gaps: confocal endomicroscopy showed increased density of small bowel epithelial gaps in inflammatory bowel disease. J Clin Gastroenterol. 2011;45 (3):240–245. 21030873 10.1097/MCG.0b013e3181fbdb8a

[46] Lobo SM De Backer D Sun QH Gut mucosal damage during endotoxic shock is due to mechanisms other than gut ischemia. J Appl Physiol. 2004;96 (2):829–829. 10.1152/japplphysiol.00925.2002 12923122

[47] Madara JL Maintenance of the macromolecular barrier at cell extrusion sites in intestinal epithelium: physiological rearrangement of tight junctions. J Membr Biol. 1990;116 (2):177–184. 2380981 10.1007/BF01868675

[48] Marchiando AM Shen L Graham WV The epithelial barrier is maintained by in vivo tight junction expansion during pathologic intestinal epithelial shedding. Gastroenterology. 2011;140 (4):1208–1218. 21237166 10.1053/j.gastro.2011.01.004 PMC3066304

[49] Marshman E Booth C Potten CS The intestinal epithelial stem cell. BioEssays. 2002;24 (1):91–98. 11782954 10.1002/bies.10028

[50] Mayhew TM Myklebust R Whybrow A Epithelial integrity, cell death and cell loss in mammalian small intestine. Histol Histopathol. 1999;14 (1):257–267. 9987670 10.14670/HH-14.257

[51] McAllister CS Lakhdari O Pineton de Chambrun G TLR3, TRIF, and caspase 8 determine double-stranded RNA-induced epithelial cell death and survival in vivo. J Immunol. 2013;190 (1):418–427. 23209324 10.4049/jimmunol.1202756 PMC3551582

[52] Meddings J What role does intestinal permeability have in IBD pathogenesis? Inflamm Bowel Dis. 2008;14 (suppl 2):S138–S139. 18816777 10.1002/ibd.20719

[53] Meyerholz DK Stabel TJ Ackermann MR Early epithelial invasion by *Salmonella enterica* Serovar Typhimurium DT104 in the swine ileum. Vet Pathol. 2002;39 (6):712–720. 12450202 10.1354/vp.39-6-712

[54] Moore R Carlson S Madara JL Villus contraction aids repair of intestinal epithelium after injury. Am J Physiol Gastrointest Liver Physiol. 1989;257 (2):G274–G283. 10.1152/ajpgi.1989.257.2.G274 2764111

[55] Myklebust R Mayhew TM Further evidence of species variation in mechanisms of epithelial cell loss in mammalian small intestine: ultrastructural studies on the reindeer (*Rangifer tarandus*) and seal (*Phoca groenlandica*). Cell Tissue Res. 1998;291 (3):513–523. 9477308 10.1007/s004410051021

[56] Nelson DE Ihekwaba AE Elliott M Oscillations in NF-kappaB signaling control the dynamics of gene expression. Science. 2004;306 (5696):704–708. 15499023 10.1126/science.1099962

[57] Nikitas G Deschamps C Disson O Transcytosis of *Listeria* *monocytogenes* across the intestinal barrier upon specific targeting of goblet cell accessible E-cadherin. J Exp Med. 2011;208 (11):2263–2277. 21967767 10.1084/jem.20110560 PMC3201198

[58] Pendyala S Walker JM Holt PR A high-fat diet is associated with endotoxemia that originates from the gut. Gastroenterology. 2012;142(5):1100–1101.e1102. 22326433 10.1053/j.gastro.2012.01.034 PMC3978718

[59] Pentecost M Otto G Theriot JA Listeria monocytogenes invades the epithelial junctions at sites of cell extrusion. PLoS Pathog. 2006;2 (1):e3. 16446782 10.1371/journal.ppat.0020003 PMC1354196

[60] Peschon JJ Torrance DS Stocking KL TNF receptor-deficient mice reveal divergent roles for p55 and p75 in several models of inflammation. J Immunol 1998;160(2):943–952. 9551933

[61] Pfeffer K Matsuyama T Kundig TM Mice deficient for the 55 kd tumor necrosis factor receptor are resistant to endotoxic shock, yet succumb to *L. monocytogenes* infection. Cell. 1993;73 (3):457–467. 8387893 10.1016/0092-8674(93)90134-c

[62] Piguet PF Vesin C Guo J TNF-induced enterocyte apoptosis in mice is mediated by the TNF receptor 1 and does not require p53. Eur J Immunol. 1998;28 (11):3499–3505. 9842892 10.1002/(SICI)1521-4141(199811)28:11<3499::AID-IMMU3499>3.0.CO;2-Q

[63] Potten CS A comprehensive study of the radiobiological response of the murine (BDF1) small intestine. Int J Radiat Biol. 1990;58 (6):925–973. 1978853 10.1080/09553009014552281

[64] Rera M Clark RI Walker DW Intestinal barrier dysfunction links metabolic and inflammatory markers of aging to death in Drosophila. Proc Natl Acad Sci USA. 2012;109 (52):21528–21533. 23236133 10.1073/pnas.1215849110 PMC3535647

[65] Ritsma L Ellenbroek SIJ Zomer A Intestinal crypt homeostasis revealed at single-stem-cell level by in vivo live imaging. Nature. 2014;507 (7492):362–365. 24531760 10.1038/nature12972 PMC3964820

[66] Roulis M Armaka M Manoloukos M Intestinal epithelial cells as producers but not targets of chronic TNF suffice to cause murine Crohn-like pathology. Proc Natl Acad Sci USA. 2011;108 (13):5396–5401. 21402942 10.1073/pnas.1007811108 PMC3069201

[67] Rupani B Caputo FJ Watkins AC Relationship between disruption of the unstirred mucus layer and intestinal restitution in loss of gut barrier function after trauma hemorrhagic shock. Surgery. 2007;141 (4):481–489. 17383525 10.1016/j.surg.2006.10.008

[68] Sato T Clevers H Growing self-organizing mini-guts from a single intestinal stem cell: mechanism and applications. Science. 2013;340 (6137):1190–1194. 23744940 10.1126/science.1234852

[69] Sherry B Jue DM Zentella A Characterization of high molecular weight glycosylated forms of murine tumor necrosis factor. Biochem Biophys Res Commun. 1990;173 (3):1072–1078. 2268312 10.1016/s0006-291x(05)80895-2

[70] Shi S Wang H Gao H Increased gap density predicts weakness of the epithelial barrier in vivo by confocal laser endomicroscopy in indomethacin-induced enteropathy. Dig Dis Sci. 2014;59 (7):1398–1405. 24573719 10.1007/s10620-014-3076-8

[71] Shirkey TW Siggers RH Goldade BG Effects of commensal bacteria on intestinal morphology and expression of proinflammatory cytokines in the gnotobiotic pig. Exp Biol Med (Maywood). 2006;231 (8):1333–1345. 16946402 10.1177/153537020623100807

[72] Sodhi C Levy R Gill R DNA attenuates enterocyte Toll-like receptor 4-mediated intestinal mucosal injury after remote trauma. Am J Physiol Gastrointest Liver Physiol. 2011;300 (5):G862–G873. 21233273 10.1152/ajpgi.00373.2010 PMC3094143

[73] Song HL Lu S Liu P Tumor necrosis factor-alpha induces apoptosis of enterocytes in mice with fulminant hepatic failure. World J Gastroenterol. 2005;11 (24):3701–3709. 15968724 10.3748/wjg.v11.i24.3701 PMC4316020

[74] Song J Wolf SE Herndon DN Second hit post burn increased proximal gut mucosa epithelial cells damage. Shock. 2008;30 (2):184–188. 18197149 10.1097/SHK.0b013e318162a3f6 PMC7859870

[75] Steinbrecher KA Harmel-Laws E Sitcheran R Loss of epithelial RelA results in deregulated intestinal proliferative/apoptotic homeostasis and susceptibility to inflammation. J Immunol. 2008;180 (4):2588–2599. 18250470 10.4049/jimmunol.180.4.2588

[76] Suzuki Y Mori K Iwanaga T Intraepithelial gamma delta T cells are closely associated with apoptotic enterocytes in the bovine intestine. Arch Histol Cytol. 1997;60 (4):319–328. 9412736 10.1679/aohc.60.319

[77] Taylor CT Dzus AL Colgan SP Autocrine regulation of epithelial permeability by hypoxia: role for polarized release of tumor necrosis factor alpha. Gastroenterology. 1998;114 (4):657–668. 9516386 10.1016/s0016-5085(98)70579-7

[78] van der Flier LG Clevers H Stem cells, self-renewal, and differentiation in the intestinal epithelium. Annu Rev Physiol. 2009;71 (1):241–260. 18808327 10.1146/annurev.physiol.010908.163145

[79] van Es JH Clevers H Paneth cells. Curr Biol. 20 14;24 (12):R547–R548. 24937274 10.1016/j.cub.2014.04.049

[80] Vandenbroucke RE Dejonckheere E Van Hauwermeiren F Matrix metalloproteinase 13 modulates intestinal epithelial barrier integrity in inflammatory diseases by activating TNF. EMBO Mol Med. 2013;5 (7):1000–1016. 10.1002/emmm.201202100 PMC3721470 23723167

[81] Vollmar B Menger M Intestinal ischemia/reperfusion: microcirculatory pathology and functional consequences. Langenbecks Arch Surg. 2011;396 (1):13–29. 21088974 10.1007/s00423-010-0727-x

[82] Wang F Wang F Zou Z Active deformation of apoptotic intestinal epithelial cells with adhesion-restricted polarity contributes to apoptotic clearance. Lab Invest. 2011;91 (3):462–471. 21042290 10.1038/labinvest.2010.182

[83] Watson AJ Chu S Sieck L Epithelial barrier function in vivo is sustained despite gaps in epithelial layers. Gastroenterology. 2005;129 (3):902–912. 16143130 10.1053/j.gastro.2005.06.015

[84] Williams JM Duckworth CA Watson AJ A mouse model of pathological small intestinal epithelial cell apoptosis and shedding induced by systemic administration of lipopolysaccharide. Dis Model Mech. 2013;6 (6):1388–1399. 24046352 10.1242/dmm.013284 PMC3820262

[85] Wu S Rhee KJ Zhang M Bacteroides fragilis toxin stimulates intestinal epithelial cell shedding and gamma-secretase-dependent E-cadherin cleavage. J Cell Sci. 2007;120 (pt 11):1944–1952. 17504810 10.1242/jcs.03455 PMC3056613

